# Routine magnifying endoscopy mode improved the diagnostic efficacy of opportunistic screening for early upper gastrointestinal neoplasms: a prospective, randomized controlled study

**DOI:** 10.1186/s12876-026-04729-1

**Published:** 2026-03-11

**Authors:** Yi Li, Min Li, Lili Ren, Fucheng Zhang, Jinling Cheng, Daocong Ren, Pengcheng Guo, Jiayi Wang, Zhi Wei

**Affiliations:** 1 Department of Gastroenterology, Shandong Second Provincial General Hospital, Jinan, China; 2 Department of Financial Mathematics, Shandong University of Finance and Ecomomics, Jinan, China

**Keywords:** Early neoplasms, Routine magnifying endoscopy mode, White light endoscopy mode, Upper gastrointestinal cancer screening, Opportunistic screening

## Abstract

**Objective:**

White light endoscopy (WLE) is an important method for upper gastrointestinal cancer screening. However, studies have demonstrated that magnifying endoscopy with image-enhanced endoscopy (ME-IEE) achieves a significantly higher detection rate of early cancer compared with WLE. Current research has primarily focused on evaluating the diagnostic performance of ME-IEE, while evidence regarding its feasibility as a routine opportunistic screening mode for early upper gastrointestinal cancer in daily clinical practice remains limited.

**Methods:**

A prospective, single-center, randomized controlled trial was conducted between October 15, 2023, and October 7, 2024. Patients aged ≥ 18 years who underwent painless anesthesia esophagogastroduodenoscopy(EGD) at our endoscopy center were enrolled. Recruited patients were randomly assigned to either the routine magnifying endoscopy group (r-ME group) or the white light endoscopy group (WLE group). All suspicious lesions were biopsied. The primary endpoint was the detection rate of early neoplasms; secondary endpoints included biopsy implementation rate, biopsy positivity rate, mean screening time, and mean number of biopsies.

**Results:**

A total of 3,000 patients were randomized (1:1). The detection rate of early neoplasms and biopsy positivity rate were significantly higher in the r-ME group than the WLE group (3.87% vs. 2.27% and 6.34% vs. 3.58%, respectively; both *P* < 0.05). However, the mean screening time was slightly longer in the r-ME group, by less than a minute (643s vs. 600s, *P* < 0.05). Among expert endoscopists, the detection rate of early neoplasms was significantly higher than that of non-expert endoscopists (4.54% vs. 2.50%, *P* < 0.05), while the biopsy submission rate was significantly lower (56.5% vs. 64.4%, *P* < 0.05). For non-expert endoscopists, both the detection rate of early neoplasms and biopsy positivity rate were significantly higher in the r-ME group than in the WLE group (3.32% vs. 1.67% and 5.29% vs. 2.53%, respectively; both *P* < 0.05). In contrast, no significant differences were observed of the above endpoints between the two groups for expert endoscopists (5.28% vs. 3.81% and 9.36% vs. 6.72%, both *P* > 0.05). The detection rate of early cancer using WLE mode was significantly lower for non-expert endoscopists compared with expert endoscopists (1.67% vs. 3.81%, *P* < 0.05). Notably, when r-ME mode was used, the detection rate achieved by non-expert endoscopists did not differ significantly from that of expert endoscopists (3.32% vs. 5.28%, *P* > 0.05), nor from that of expert endoscopists using WLE mode (3.32% vs. 3.81%, *P* > 0.05).

**Conclusion:**

The r-ME model enhances diagnostic accuracy and consistency in opportunistic screening for early upper gastrointestinal cancer, offering particular value for non-expert endoscopists.

**Trial registration:**

This study was registered in the Chinese Clinical Trial Registry on 03/10/2023 (clinical trial registration number: ChiCTR2300076327).

**Supplementary Information:**

The online version contains supplementary material available at 10.1186/s12876-026-04729-1.

## Introduction

Esophageal and gastric cancers remain major public health burdens in China, despite recent declines in incidence and mortality [1]. Compared with other countries, rates remain disproportionately high [2]. Because early-stage disease is typically asymptomatic, endoscopic screening is critical for timely detection. National programs in Japan and South Korea—where participation rates reach nearly 75%—have enabled early detection in approximately half of gastric cancer cases, with five-year survival rates exceeding 64% [3–5].In contrast, although China’s nationwide screening programs have improved survival, outcomes remain markedly inferior [1]. Expanding strategies for early detection in the general population and targeted high-risk groups is therefore essential. Given the impracticality of universal screening, opportunistic screening has emerged as a pragmatic and effective alternative. White light endoscopy (WLE) is the standard modality for opportunistic screening of upper gastrointestinal cancers. However, magnifying endoscopy with image-enhanced endoscopy (ME-IEE) allows detailed visualization of microvascular and microsurface structures, significantly improving early cancer detection compared with WLE [6, 7]. Beyond enhanced diagnostic accuracy, ME-IEE may facilitate earlier intervention, improved prognosis, and reduced healthcare burden [8, 9]. Yet, its potential as a routine tool for opportunistic screening remains underexplored, and evidence from China is scarce.

## Methods

### Study subjects

This study was a prospective, single center, randomized controlled trial, and was conducted in accordance with the Declaration of Helsinki and the guidelines of the Consolidated Standards of Reporting Trials (CONSORT). The study protocol was approved by the Institutional Ethics Board of the Shandong Second Provincial General Hospital, Jinan, China (No. 2023-021-01) and registered in the Chinese Clinical Trial Registry (No: ChiCTR2300076327). Enrollment between October 10, 2023, and October 7, 2024.

### Inclusion and exclusion criteria

**Fig. 1:**
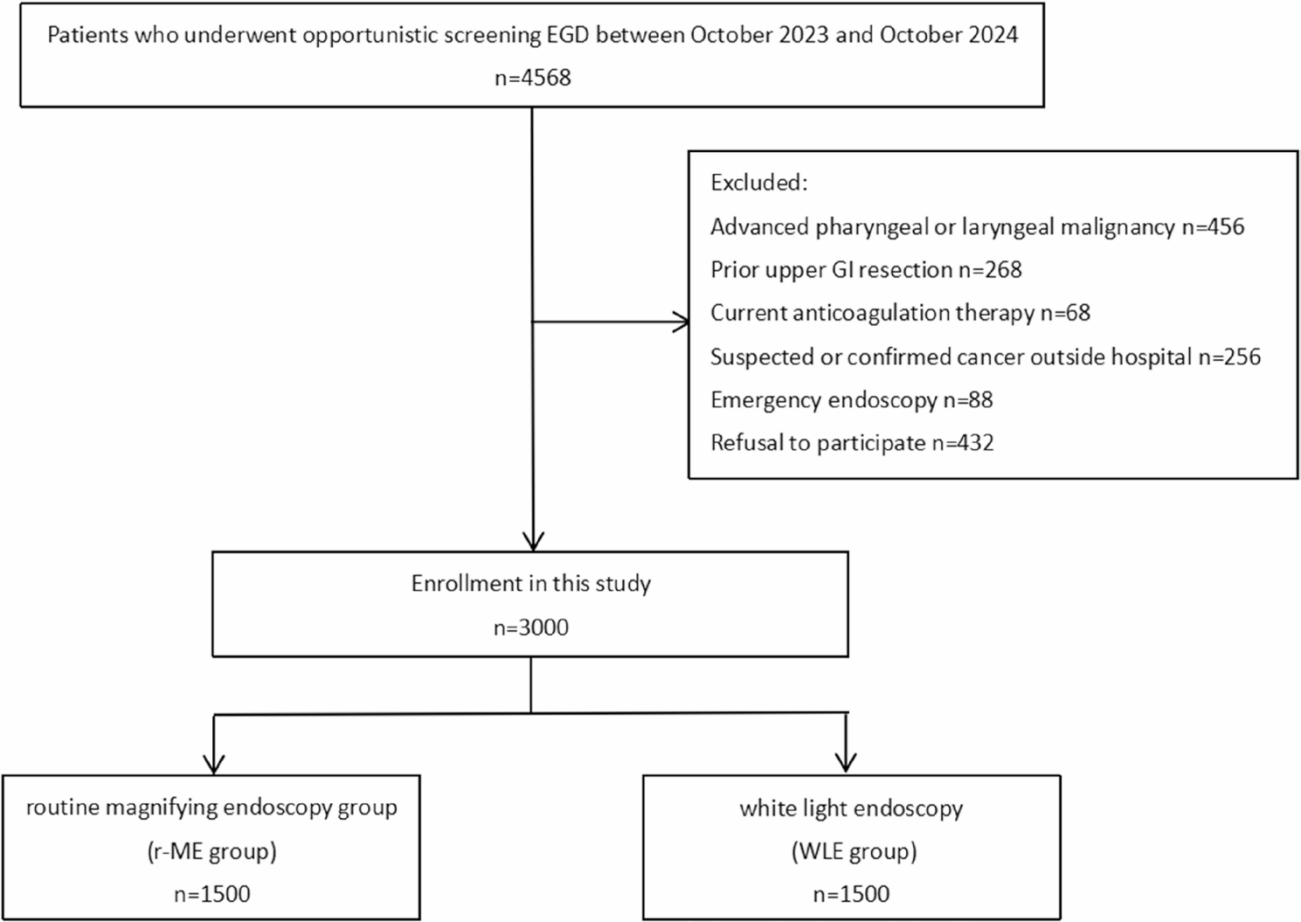
A flow diagram shows the participant selection and grouping in this study

According to medical history data from other hospitals, the detection rate of early cancer was 2.57% in the r-ME group and 0.25% in the WLE group. The significance level was set at α = 0.05 and β = 0, with a refusal rate of 10%. Based on sample size calculation, the minimum sample size for each group was 673. A total of 4,568 patients undergoing painless EGD for opportunistic screening were screened, of whom 3,000 met eligibility criteria. A random number table was generated according to a 1:1 allocation ratio between the control group and the experimental group.Patients were randomized by physicians based on the random number table into the r-ME group and the WLE group using a single-blind design (patient-blinded). Exclusion criteria included advanced pharyngeal or laryngeal malignancy, prior upper gastrointestinal resection, current antithrombotic therapy, suspected or confirmed cancer at another institution, emergency endoscopy, or refusal to participate (Fig. 1).

### Study methods

Before EGD, all enrolled patients completed the basic information in the Case Report Form (CRF), including sex, age, height, weight, body mass index (BMI), high-temperature diet, high-salt diet, heavy smoking, heavy alcohol consumption, alcohol flushing reaction, comorbidities, upper gastrointestinal neoplasms family history, allergy history, precancerous gastric conditions, Helicobacter pylori (Hp) infection history, Hp eradication history, and proton pump inhibitor (PPI) use within the past week.Physicians then recorded the group allocation and the endoscope to be used. After the examination, physicians recorded the biopsy sites, primary diagnosis, and the start and end times of the procedure based on when the endoscope first passed through and finally left the pharynx. Cases were marked as “complete” once the corresponding pathological results were confirmed; those lost to follow-up were marked as “dropout”.

In the r-ME group, WLE was performed initially, followed by ME-IEE when suspicious lesions were detected to guide targeted biopsy. In the WLE group, only WLE was performed, with biopsies taken when suspicious lesions were identified. Histopathology was used as the reference standard. The primary endpoint was the detection rate of early neoplasms; secondary endpoints included biopsy implementation rate, biopsy positivity rate, mean screening time, and mean number of biopsies. In this study, diagnostic criteria during magnifying endoscopy with IEE (only NBI) were based on the evaluation of mucosal micro-surface structure and microvascular architecture. Abnormalities including irregular, distorted or absent microsurface patterns, dilated, tortuous, heterogeneous or irregular microvessels, and distinct demarcation lines between the lesion and adjacent normal mucosa were regarded as highly suggestive of precancerous lesions or early upper gastrointestinal neoplasms. Biopsy was performed when lesions showed any of the following features: (1) suspicious mucosal color, elevation, depression, or erosion under white-light endoscopy; (2) atypical microsurface or microvascular patterns under magnifying IEE; (3) lesions highly suspicious of inflammation, precancerous conditions, or early neoplasia; (4) lesions with unclear diagnosis requiring pathological confirmation.

### Endoscopic and pathological assessment

Olympus endoscopes (models H260, H290, Q260J, H260Z, and H290Z) were used. All biopsy specimens were independently reviewed by two senior pathologists and classified according to the Vienna classification as non-neoplastic, low-grade dysplasia (LGD), high-grade dysplasia (HGD), squamous cell carcinoma (SCC), or adenocarcinoma(AC).

### Statistical analysis

Analyses were performed using SPSS version 25.0 and Python 3.9.13. Continuous variables were compared with t-tests or nonparametric tests, and categorical variables with chi-square or Fisher’s exact tests, as appropriate. All tests were two-tailed, with *P* < 0.05 considered statistically significant.

### Definitions

High temperature diet was defined as consumption of hot food or beverages at a temperature ≥ 65 °C at least 3 times per week. High salt diet was defined as an average daily salt intake ≥ 5 g per day, or frequent consumption of pickled, smoked, or salted foods. Heavy smoking was defined as smoking ≥ 20 cigarettes per day for ≥ 5 years. Heavy alcohol consumption was defined as daily alcohol intake exceeding 40 g of pure alcohol for at least 1 year. Alcohol flushing reaction was defined as visible facial flushing or redness shortly after alcohol intake. Comorbidities included hypertension, coronary heart disease, and diabetes mellitus. Precancerous gastric conditions included chronic atrophic gastritis, intestinal metaplasia, dysplasia, gastric ulcer, and gastric polyps. Suspicious lesions in the upper gastrointestinal tract were defined as mucosal areas with abnormal color, irregular surface, depressed or elevated morphology, distortion of microsurface structure, or abnormal microvascular architecture as observed under endoscopy, which were highly suspected of inflammation, precancerous lesions, or early neoplasia. Expert endoscopists were those who had performed at least 500 magnifying endoscopy procedures and had more than 5 years of clinical experience in diagnosing early upper gastrointestinal neoplasms. Detection rate was defined as the percentage of patients who receive a positive diagnosis to total biopsies.

## Results

### Baseline characteristics

A total of 3,000 patients were enrolled and randomized equally into the r-ME group (*n* = 1500) and the WLE group (*n* = 1500). Baseline characteristics, including sex, age, height, weight, body mass index (BMI), high-temperature diet, high-salt diet, heavy smoking, heavy alcohol consumption, alcohol flushing reaction, comorbidities, family history, allergy history, precancerous gastric conditions, Helicobacter pylori (Hp) infection history, Hp eradication history, and proton pump inhibitor (PPI) use within the past week, did not differ significantly between the two groups (all *P* > 0.05) (Table 1).

**Table 1:** The baseline characteristics of the patients in the r-ME group and WLE group

	Total *N* = 3000	WLE group *N* = 1500	*r*-ME group *N* = 1500	*P* value
Sex				1.000
Female	1473 (49.1%)	737 (49.1%)	736 (49.1%)	
Male	1527 (50.9%)	763 (50.9%)	764 (50.9%)	
Age(y) (Mean ± SD)	52.9 ± 13.0	52.7 ± 13.1	53.1 ± 12.9	0.361
Height(m) (Mean ± SD)	1.72 ± 2.98	1.66 ± 0.08	1.77 ± 4.22	0.330
Weight(kg) (Mean ± SD)	68.0 ± 12.3	67.9 ± 12.4	68.1 ± 12.3	0.593
BMI(kg/m^2^) (Mean ± SD)	24.5 ± 3.62	24.4 ± 3.58	24.6 ± 3.66	0.236
High-temperature diet				0.448
No	2251 (75.0%)	1135 (75.7%)	1116 (74.4%)	
Yes	749 (25.0%)	365 (24.3%)	384 (25.6%)	
High-salt diet				0.935
No	2167 (72.2%)	1085 (72.3%)	1082 (72.1%)	
Yes	833 (27.8%)	415 (27.7%)	418 (27.9%)	
Heavy smoking				0.692
No	2513 (83.8%)	1261 (84.1%)	1252 (83.5%)	
Yes	487 (16.2%)	239 (15.9%)	248 (16.5%)	
Heavy alcohol consumption				0.776
No	2457 (81.9%)	1232 (82.1%)	1225 (81.7%)	
Yes	543 (18.1%)	268 (17.9%)	275 (18.3%)	
Alcohol flushing reaction				0.964
No	404 (73.5%)	202 (73.7%)	202 (73.2%)	
Yes	146 (26.5%)	72 (26.3%)	74 (26.8%)	
Comorbidities				0.663
No	2317 (77.2%)	1164 (77.6%)	1153 (76.9%)	
Yes	683 (22.8%)	336 (22.4%)	347 (23.1%)	
Family history				0.342
No	2636 (87.9%)	1309 (87.3%)	1327 (88.5%)	
Yes	364 (12.1%)	191 (12.7%)	173 (11.5%)	
Allergy history				0.220
No	2819 (94.0%)	1401 (93.4%)	1418 (94.5%)	
Yes	181 (6.0%)	99 (6.6%)	82 (5.5%)	
Precancerous gastric conditions				0.325
No	2440 (81.3%)	1209 (80.6%)	1231 (82.1%)	
Yes	560 (18.7%)	291 (19.4%)	269 (17.9%)	
Hp infection history				0.112
unknow	1134 (37.8%)	555 (37.0%)	579 (38.6%)	
No	1423 (47.4%)	738 (49.2%)	685 (45.7%)	
Yes	443 (14.8%)	207 (13.8%)	236 (15.7%)	
Hp eradication history				0.165
No	2723 (90.8%)	1373 (91.5%)	1350 (90.0%)	
Yes	277 (9.2%)	127 (8.5%)	150 (10.0%)	
PPI use within the past week				0.461
No	2877 (95.9%)	1434 (95.6%)	1443 (96.2%)	
Yes	123 (4.1%)	66 (4.4%)	57 (3.8%)	

### Early neoplasms detection rates

In the r-ME group, 66 early neoplasms were detected (1 pharyngeal, 41 esophageal, 20 gastric, and 4 duodenal), whereas 40 lesions were identified in the WLE group (2 pharyngeal, 23 esophageal, 13 gastric, and 2 duodenal) (Table 2). The early neoplasms detection rate was significantly higher in the r-ME group compared with the WLE group (3.87% [58/1,500] vs. 2.27% [34/1,500], *P* < 0.05) (Table 3).

**Table 2:** The early neoplasms number detected in different parts of the r-ME group and WLE group

	Pharynx(*n*)	Esophagus(*n*)	stomach(*n*)	duodenum(*n*)	Total(*n*)
*r*-ME group	1(1.52%)	41(62.12%)	20(30.30%)	4(6.06%)	66
WLE group	2(5.00%)	23(57.50%)	13(32.50%)	2(5.00%)	40

**Table 3:** Early neoplasms detection rates in the r-ME group and WLE group

	r-ME group	WLE group	*P* value
Patients(n)	1500	1500	0.011
Early neoplasms patients numbers(n)	58	34	
Early neoplasms detection rates(%)	3.87%	2.27%	

### Biopsy performance and mean screening time

Biopsy implementation rate and mean number of specimens were similar between the two groups( 61.3% vs. 63.6%, 1.95 vs. 2.01, respectively; both *P*>0.05), whereas biopsy positivity rate was higher in the r-ME group (6.34% [58/915] vs. 3.58% [34/950], *P* < 0.05). Mean examination time was slingtly longer for the r-ME group (643 s vs. 600 s, *P* < 0.05) (Table 4).

**Table 4:** Biopsy performance and mean screening time in the r-ME group and WLE group

	*r*-ME group	WLE group	*P* value
Patients(*n*)	1500	1500	
Biopsy patients(n)	915	950	
Biopsy Implementation rate(%)	61.3	63.6	0.188
Mean number of biopsies(n)	1.95	2.01	0.691
Biopsy positivity rate(%)	6.34	3.58	0.006
Mean screening time(s)	643	600	*P* < 0.001

### Comparison between expert endoscopists and non-expert endoscopists

Four expert endoscopists performed 837 endoscopic screenings (27.9%) and six non-expert endoscopists 2,163 screenings(72.1%). Early neoplasms detection rates was higher among expert endoscopists (4.54% vs. 2.50%, *P* < 0.05), while biopsy submission rate was lower (56.5% vs. 64.4%, *P* < 0.05). No difference was observed in mean number of biopsy specimens (2.02 vs. 1.97, *P* > 0.05). And mean screening time was shorter for expert endoscopists (589s vs. 634s, *P* < 0.05) (Table 5).

**Table 5:** Comparison between expert endoscopists and non-expert endoscopists

	Total *N* = 3000	expert endoscopists *N* = 837	non-expert endoscopists *N* = 2163	*P* value
Groups				0.935
WLE group	1500 (50.0%)	420 (50.2%)	1080 (49.9%)	
r-ME group	1500 (50.0%)	417 (49.8%)	1083 (50.1%)	
Detect early neoplasms(n)				0.020
No	2908 (96.93%)	799 (95.46%)	2109 (97.50%)	
Yse	92 (3.07%)	38 (4.54%)	54 (2.50%)	
Biopsy submission(%)				< 0.001
No	1135 (37.8%)	365 (43.6%)	770 (35.6%)	
Yes	1865 (62.2%)	473 (56.5%)	1392 (64.4%)	
Mean number of biopsies(n)	1.98	2.02	1.97	0.544
Mean screening time (s)	621	589	634	< 0.001

### Comparison of endoscopy modes among expert endoscopists

Among expert endoscopists, mean screening time was longer in the r-MEmode compared in the WLE mode (611s vs. 568s, *P* < 0.05), but early neoplasms detection rate, biopsy implementation rate, biopsy positivity rate, and mean number of biopsies did not differ significantly between the two modes (all *P* > 0.05) (Table 6).

**Table 6:** Comparison of endoscopy modes among expert endoscopists

	*r*-ME mode	WLE mode	*P* value
Patients (*n*)	417	420	
Biopsy patients(n)	235	238	
Biopsy implementation rate(%)	56.4	56.7	0.928
Mean number of biopsies(n)	1.98	2.05	0.375
Early neoplasms patients numbers(n)	22	16	
Early neoplasms detection rates(%)	5.28	3.81	0.308
Biopsy positivity rate(%)	9.36	6.72	0.291
Mean screening time(s)	611	568	0.032

### Comparison of endoscopy modes among non-expert endoscopists

Among non-expert endoscopists, r-ME mode significantly increased early neoplasms detection rate (3.32% vs. 1.67%) and biopsy positivity rate (5.29% vs. 2.53%) compared with WLE mode(*P* < 0.05), with a modest increase in screening time (655s vs. 612s, *P* < 0.05). Biopsy implementation rate and mean number of biopsies did not differ significantly between the two modes (both *P* > 0.05) (Table 7).

**Table 7:** Comparison of endoscopy modes among non-expert endoscopists

	*r*-ME mode	WLE mode	*P* value
Patients(*n*)	1083	1080	
Biopsy patients(n)	680	712	
Biopsy implementation rate(%)	62.8	65.9	0.128
Mean number of biopsies(n)	1.94	2.00	0.927
Early neoplasms patients numbers(n)	36	18	
Early neoplasms detection rates(%)	3.32	1.67	0.013
Biopsy positivity rate(%)	5.29	2.53	0.008
Mean screening time(s)	655	612	< 0.001

### Cross-comparison between expert and non-expert endoscopists under different modes

With WLE mode, the early neoplasms detection rate was significantly lower for non-expert endoscopists compared with expert endoscopists (1.67% vs. 3.81%, *P* < 0.05). Using r-ME mode, non-expert endoscopists detection rates did not differ significantly from expert using r-ME mode (3.32% vs. 5.28%, *P* > 0.05) or from expert using WLE mode (3.32% vs. 3.81%, *P* > 0.05). Detection rate was lowest for non-expert endoscopists using WLE mode compared with expert using r-ME mode(1.67% vs. 5.28%, *P* < 0.05) (Table 8).

**Table 8:** Cross-comparison between expert and non-expert endoscopists under different modes

	Detect no early neoplasms (*n*)	Detect early neoplasms (*n*)	Total (*n*)	Early neoplasms detection rates (%)	*P* value
Expert endoscopists(WLE mode)	404	16	420	3.81	0.021
Non-expert endoscopists(WLE mode)	1062	18	1080	1.67	
Total(n)	1466	34	1500		
Expert endoscopists(r-ME mode)	395	22	417	5.28	0.108
Non-expert endoscopists(r-ME mode)	1047	36	1083	3.32	
Total(n)	1442	58	1500		
Expert endoscopists(WLE mode)	404	16	420	3.81	0.761
Non-expert endoscopists (r-ME mode)	1047	36	1083	3.32	
Total(n)	1451	52	1503		
Expert endoscopists (r-ME mode)	395	22	417	5.28	< 0.001
Non-expert endoscopists (WLE mode)	1062	18	1080	1.67	
Total(n)	1457	40	1497		

## Discussion

Endoscopic screening remains the most effective approach for early detection of upper gastrointestinal (GI) tumors. In a large Chinese cohort (637,500 participants), endoscopic screening reduced upper GI tumor incidence by 23% (RR = 0.77, 95% CI: 0.74–0.81) and mortality by 57% (RR = 0.43, 95% CI: 0.40–0.47) [10]. In patients with invasive gastric cancer, overall survival and disease-specific survival were significantly higher in individuals who underwent screening, particularly in those who received repeated screening [10]. ME plays an important role in the qualitative diagnosis and assessment of invasion depth for superficial squamous cell carcinoma of the esophagus and pharynx [11–13]. Studies have shown that both non-ME and ME-NBI could improve detection rates of superficial esophageal squamous cell carcinoma in high-risk populations [11, 12]. NBI could also enhance detection of superficial pharyngeal squamous cell carcinoma [13]. In high-risk patients, ME-NBI achieves a diagnostic accuracy of 91.2% (95% CI: 85.4–95.2) for esophageal squamous cell carcinoma and high-grade intraepithelial neoplasia [14]. ME is particularly valuable for the qualitative diagnosis of early gastric cancer, especially flat lesions and small gastric cancers [15, 16], and can assist in evaluating the background gastric mucosa [17], enabling precise diagnosis of precancerous conditions such as intestinal metaplasia and dysplasia [18]. Although many studies suggest that ME may improve detection of upper GI tumors, its routine application remains debated.The present study adds to the literature by providing prospective, randomized evidence supporting the clinical feasibility of this approach in opportunistic screening.

A Japanese retrospective study of opportunistic screening found the ME group demonstrated a significantly higher detection rate of gastric intraepithelial neoplasia (2.3% vs. 1.3%, *P* = 0.023), although the difference in gastric cancer detection was not statistically significant (1.6% vs. 1.0%, *P* = 0.162) [19]. Similarly, a domestic study reported that ME detected early upper GI cancers at a rate of 3.70% (303/8,184), significantly higher than 1.43% (345/24,183) by WLE [16]. Our study showed early upper GI neoplasms detection rates of 3.87% for ME and 2.27% for WLE, consistent with previous findings, supporting the role of ME in improving early neoplasms detection. However, another Japanese study [20] demonstrated no significant difference in the detection rate of epithelial neoplasms between the magnified and non-magnified groups (0.8% vs. 0.3%; *P* = 0.14). The investigators hypothesized that this finding might be associated with three factors: the study population was characterized by a low prevalence of upper gastrointestinal cancer; both groups initially adopted non-magnified observation to identify suspected epithelial neoplastic lesions; and iodine staining in the esophagus might abrogate the differences in detection and biopsy rates. Notably, the detection rate of epithelial neoplasms in the magnified group (0.8%) was more than twice that in the non-magnified group (0.3%). Based on this observation, the authors concluded that the application of magnification endoscopy (ME) facilitates the avoidance of tumor missed diagnosis and may potentially improve the detection rate of gastrointestinal epithelial neoplasms compared with non-ME. In our study, the r‑ME group detected a greater number of esophageal epithelial neoplasms (41 lesions, 62.12%). However, iodine staining was not performed as a complementary examination in our study, which may have contributed to the observed difference in detection rates between the two groups.

ME also provides a degree of “optical biopsy”. A prospective multicenter study reported that high-confidence ME-NBI diagnosis achieved an accuracy of 98.1%, sensitivity of 85.7%, and specificity of 99.4% [21]. Excluding whitish mucosal lesions further increased these metrics to 99.4%, 100%, and 99.4%, suggesting that high-confidence ME-NBI may obviate the need for biopsy in certain lesions [21]. A domestic study found that ME-targeted biopsy (MNTB) had higher sensitivity than endoscopic forceps biopsy (EFB) (*P* = 0.048), while combined ME-NBI did not significantly differ in sensitivity, specificity, or accuracy between the two methods [22]. Japanese studies demonstrated that ME reduced biopsy rates and increased positive predictive value compared to non-ME [19, 20]. These results indicate that ME screening mode may improve biopsy efficiency and reduces unnecessary tissue sampling. Our findings similarly showed that although biopsy rates were comparable between r-ME and WLE groups, ME significantly increased biopsy positivity (6.34% vs. 3.58%, *P* < 0.05) and prolonged examination time modestly (643 s vs. 600 s, *P* < 0.05). Compared with foreign countries, our tissue biopsy rates had not decreased, but there was no significant difference in positive predictive value, suggesting that the reduction of biopsies achieved through the application of ME may depend on the training of endoscopists and changes in diagnostic habits. Although examination time was slightly longer in the magnifying group, the clinical benefit of improved detection outweighs this limitation. With further refinement of endoscopic training and workflow optimization, the time difference may be minimized in routine practice.

Physician experience could also influence early cancer detection. Expert endoscopists achieved higher early cancer detection rates, performed fewer biopsies, and required shorter examination times compared to non-expert endoscopists. Notably, expert endoscopists detection rates were less dependent on endoscopy modes, whereas non-expert endoscopists benefitted significantly from r-ME mode, achieving detection rates comparable to expert. This suggests that r-ME can reduce the performance gap among physicians of varying experience levels, particularly enhancing non-expert endoscopists’ detection capabilities. Previous studies also indicate that standardized ME diagnostic frameworks can narrow the diagnostic gap between expert and non-expert endoscopists [20, 23].

Recent Chinese consensus guidelines endorse routine ME combined with pathology for early upper GI cancer screening [24]. Our findings provided clinical evidence supporting its implementation. The present study has several strengths. First, it is a large-scale, prospective, randomized controlled trial directly comparing magnifying endoscopy with WLE in a real-world clinical screening setting. Second, subgroup analyses by operator experience provide novel insights into how magnifying endoscopy may reduce inter-operator variability and serve as a valuable training tool for junior endoscopists.

In this prospective randomized controlled trial, the diagnostic performance of routine magnifying endoscopy was compared with that of WLE in the opportunistic screening of early upper gastrointestinal neoplasms. The key finding of this study was that r-ME mode significantly improved the early neoplasms detection rate and biopsy positivity rate, albeit with a modest increase in examination time. Importantly, magnifying endoscopy was shown to narrow the gap in diagnostic performance between junior and senior endoscopists, particularly enhancing the detection rate of junior endoscopists. Nevertheless, several limitations should be acknowledged. This was a single-center study, which may limit the generalizability of the findings to other populations. In the present study, we only determined the pathological nature of the lesions. We did not analyze the diagnostic efficacy with respect to lesion size, gross classification, histological type, or depth of invasion. Absence of follow-up, and inability to assess missed lesions were not assessed and warrant further investigation. Future multicenter randomized trials are needed to confirm the value of routine ME in early upper GI cancer opportunistic screening.

## Conclusion

Routine ME mode demonstrated clear advantages in the detection of early upper gastrointestinal cancers compared with WLI. Our findings confirmed that its use significantly increased early cancer detection rates and biopsy positivity, albeit at the cost of slightly prolonged examination time. Importantly, magnifying endoscopy appeared to mitigate disparities in diagnostic performance between senior and junior endoscopists, as the detection rates of less experienced physicians improved markedly with its use. These results suggest that magnifying endoscopy not only enhances diagnostic accuracy but also provides an effective strategy to standardize diagnostic quality across varying levels of endoscopist expertise. Consequently, its routine implementation in opportunistic screening may represent a critical step toward improving early detection and reducing variability in clinical outcomes.

## Supplementary Information

Supplementary Material 1.

## Data Availability

The datasets used during the current study are available from the corresponding author on reasonable request.

## Abbreviations

WLE: White light endoscopy
ME-IEE: Magnifying endoscopy with image-enhanced endoscopy
r-ME: routine-Magnifying endoscopy
EGD: Esophagogastroduodenoscopy
BMI: Body mass index
GI: Gastrointestinal
