# Supplementary material for: Routine magnifying endoscopy mode improved the diagnostic efficacy of opportunistic screening for early upper gastrointestinal neoplasms: a prospective, randomized controlled study
Source: BMC Gastroenterol. 2026 Mar 11;26:239. doi: 10.1186/s12876-026-04729-1 (PMC13093946; doi:10.1186/s12876-026-04729-1)
Supplement: Supplementary file 1 — Supplementary Material 1. [file 12876_2026_4729_MOESM1_ESM.docx]

# CONSORT 2010 Checklist of Information to Include When Reporting a Randomized Trial

Manuscript title: Routine magnifying endoscopy mode improved the diagnostic efficacy of opportunistic screening for early upper gastrointestinal neoplasms: a prospective, randomized controlled study

| Section/Topic | Item No. | Checklist item | Reported on page No. |
| --- | --- | --- | --- |
| Title and Abstract | 1a | Identification as a randomized trial in the title | 1 |
|  | 1b | Structured summary of trial design, methods, results, and conclusions | 1–2 |
| Introduction | 2a | Scientific background and explanation of rationale | 2-3 |
|  | 2b | Specific objectives or hypotheses | 3 |
| Methods - Trial design | 3a | Description of trial design (such as parallel, factorial), including allocation ratio | 3 |
|  | 3b | Important changes to methods after trial commencement (such as eligibility criteria), with reasons | Not applicable |
| Participants | 4a | Eligibility criteria for participants | 3 |
|  | 4b | Settings and locations where the data were collected | 4 |
| Interventions | 5 | The interventions for each group with sufficient details to allow replication, including how and when they were administered | 4 |
| Outcomes | 6a | Completely defined pre-specified primary and secondary outcome measures, including how and when they were assessed | 4 |
|  | 6b | Any changes to trial outcomes after the trial commenced, with reasons | Not applicable |
| Sample size | 7a | How sample size was determined | 3 |
|  | 7b | When applicable, explanation of any interim analyses and stopping guidelines | Not applicable |
| Randomization - Sequence generation | 8a | Method used to generate the random allocation sequence | 3 |
|  | 8b | Type of randomization; details of any restriction (such as blocking and block size) | 3 |
| Allocation concealment mechanism | 9 | Mechanism used to implement the random allocation sequence (such as sealed opaque envelopes), describing any steps taken to conceal the sequence until interventions were assigned | 3 |
| Implementation | 10 | Who generated the random allocation sequence, who enrolled participants, and who assigned participants to interventions | 3 |
| Blinding | 11a | If done, who was blinded after assignment to interventions (e.g., participants, care providers, outcome assessors) and how | 3 |
|  | 11b | If relevant, description of the similarity of interventions | 4 |
| Statistical methods | 12a | Statistical methods used to compare groups for primary and secondary outcomes | 4 |
|  | 12b | Methods for additional analyses, such as subgroup analyses and adjusted analyses | 4 |
| Results - Participant flow | 13a | For each group, the numbers of participants who were randomly assigned, received intended treatment, and were analyzed for the primary outcome | 4; Figure 1 |
|  | 13b | For each group, losses and exclusions after randomization, together with reasons | 4; Figure 1 |
| Recruitment | 14a | Dates defining the periods of recruitment and follow-up | 3 |
|  | 14b | Why the trial ended or was stopped | 3 |
| Baseline data | 15 | A table showing baseline demographic and clinical characteristics for each group | 5-7 |
| Numbers analyzed | 16 | For each group, number of participants (denominator) included in each analysis and whether the analysis was by original assigned groups | 5-7 |
| Outcomes and estimation | 17a | For each primary and secondary outcome, results for each group, and the estimated effect size and its precision (such as 95% CI) | 7-12; Table 2-8 |
|  | 17b | For binary outcomes, presentation of both absolute and relative effect sizes is recommended | 7-12; Table 2-8 |
| Ancillary analyses | 18 | Results of any other analyses performed, including subgroup analyses and adjusted analyses, distinguishing pre-specified from exploratory | 8-12; Table 5-8 |
| Harms | 19 | All important harms or unintended effects in each group | Not applicable |
| Discussion | 20 | Trial limitations, addressing sources of potential bias, imprecision, and, if relevant, multiplicity of analyses | 14-15 |
|  | 21 | Generalizability (external validity, applicability) of the trial findings | 13-14 |
|  | 22 | Interpretation consistent with results, balancing benefits and harms, and considering other relevant evidence | 12-14 |
| Other information | 23 | Registration number and name of trial registry | 2(ChiCTR2300076327) |
|  | 24 | Where the full trial protocol can be accessed, if available | Available upon request |
|  | 25 | Sources of funding and other support (such as supply of equipment), role of funders | 16 (No funding) |
